# The effects of a nationwide program to reduce seclusion in the Netherlands

**DOI:** 10.1186/1471-244X-12-231

**Published:** 2012-12-18

**Authors:** Fleur J Vruwink, Cornelis L Mulder, Eric O Noorthoorn, Daan Uitenbroek, Henk LI Nijman

**Affiliations:** 1 GGNet, PO Box 2003, 7230 GC, Warnsveld, the Netherlands; 2 Public Mental Health, Research Center O3, Erasmus MC, Rotterdam, the Netherlands; 3 Bavo Europoort, Mental Health Center Rotterdam, Prins Constantijnweg 48-54, 3066 TA, Rotterdam, the Netherlands; 4 GGNet, Bestuursbureau, PO Box 2003, 7230 GC, Warnsveld, the Netherlands; 5 Quantitative Skills, Consultancy for Research and Statistics, Lieven de Keylaan 7, 1222 LC, Hilversum, The Netherlands; 6 Forensic psychology, Behavioural Science Institute (BSI), Radboud University, Nijmegen, the Netherlands; 7 Altrecht Aventurijn, Dolderseweg 164, 3734 BN, Den Dolder, the Netherlands

**Keywords:** Seclusion, Restraint, Coercive measures, Reduction, Nationwide effort

## Abstract

**Background:**

From 2006 to 2009, the Dutch government provided €5 m annually for a nationwide program to reduce seclusion in psychiatric hospitals by 10% a year. We aimed to establish whether the numbers of both seclusion and involuntary medication changed significantly after the start of this national program.

**Methods:**

Using Poisson regression to estimate difference in logit slopes, we analyzed data for 1998–2009 from the Dutch Health Care Inspectorate, retrospectively examining the national numbers of seclusion and involuntary medication before and after the start of the program.

**Results:**

The difference in slopes of the numbers of seclusion before and after the start of the program was statistically significant (difference 5.2%: p < 0.001). After the start of the program seclusions dropped 2.0% per year. Corrected for the increasing number of involuntary hospitalizations this figure was 4.7% per year. The difference in slopes of the numbers of involuntary medication did not change statistically significant (difference 0.5%, n.s.). After correction for the increasing number of involuntary hospitalizations the difference turned significant (difference 3.3%, p = 0.002).

**Conclusions:**

After the start of the nationwide program the number of seclusions fell, and although significantly changing, the reduction was modest and failed to meet the objective of a 10% annual decrease. The number of involuntary medications did not change; instead, after correction for the number of involuntary hospitalizations, it increased.

## Background

The coercive intervention of first choice in most European countries is involuntary medication [1]. In the Netherlands, it is seclusion. This partly explains why the use of seclusion is much higher in the Netherlands than in surrounding countries [2,3]. To fund a nationwide program to reduce seclusion by 10% per year, the Dutch government therefore provided €5 m annually from 2006 to 2009 [4].

This retrospective study was intended to establish whether the number of seclusions had changed significantly since the start of this program. As the program focused mainly on reducing seclusion and not on other coercive interventions, our second objective was to determine whether there had been a concomitant increase in the use of involuntary medication.

## Methods

### Nationwide program

In 2006, grants were awarded to 34 Dutch psychiatric hospitals (approximately 70% of all psychiatric hospitals), a number that had increased to 42 by 2009 (approximately 90%). The grants were allocated only to psychiatric hospitals that had a specific plan how to reduce the number of seclusions [4] and would also match the sum they received. The total national investment was therefore €40 m, i.e. €20 m from the government and €20 m from the hospitals. Criteria for receiving the grant included the plan having a specific target for reducing seclusion, developing psychiatric intensive care, gathering reliable data on coercive measures, and enhancing expertise of staff (e.g. using specific strategies for preventing seclusion and dealing with problematic behaviours). The projects were very varied in scope. Some sought reductions at institutional level (e.g. closing seclusion rooms), others at ward level (e.g. through new engagement strategies or aggression de-escalation training), others at patient level (e.g. through crisis plans or aggression-risk assessment), and others by combining various levels and strategies.

### Coercive measures

Seclusion was defined as locking up a patient in a room designed for this purpose without opportunities to leave. Involuntary medication was defined as any medication administered (usually intramuscularly) against a patient’s will in cases of emergency or within enforced treatment.

In the Netherlands, all coercive interventions have to be reported to the Dutch Health Care Inspectorate (DHCI). Such measures, including seclusion and involuntary medication, are permitted only within the context of involuntary hospitalizations, which thereby defines the population at risk. An involuntary hospitalization can be requested for both inpatients and outpatients, when the patient causes danger to himself or others, caused by the psychiatric illness.

The DHCI provided quarterly numbers of seclusion, involuntary medication and involuntary hospitalizations from 1998 until 2009.

### Analyses

We analysed changes in absolute and corrected quarterly numbers of seclusion and involuntary medication. Because the number of involuntary hospitalizations increased in the Netherlands in this period [5], we corrected our analyses for the number of involuntary hospitalizations by standardizing the analyses of change to a fixed number of involuntary hospitalizations.

A Poisson regression in the R statistical package (http://www.r-project.org) was used to compare the slopes of the logit lines before and after the start of the nationwide program in 2006. The relevant equation being: *count of seclusions* = *exp*[*intercept* + slope1*quartile-*number* + slope2*quartile-number*program], with program coded as 0 before the start of the program in 2006 and 1 afterwards. Slope1 is the exponential slope before 2006. Slope2 is the difference between slope1 and the exponential slope since 2006. To aid interpretation, the slopes are presented in annual percentages of increase or decrease. Analyses were performed on quarterly data.

## Results

### Raw numbers

Whereas the number of seclusions had increased 3.2% annually from 1998 to 2005 (logit slope = 1.032), they fell significantly between 2006 and 2009 to an annual decrease of 2.0% (logit slope = 0.980, difference −5,2%: z = −8.58, p < 0.001).

**Figure 1:**
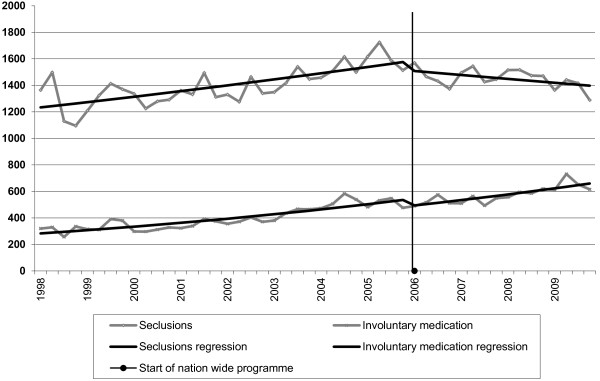
**National numbers of seclusion**/**involuntary medication before and after the start of a nationwide program.** Total number of seclusions and involuntary medications in the Netherlands per quarter, before and after the start of a nationwide program in 2006 to reduce the use of seclusion, both in absolute numbers and using a Poisson regression line. The slope of the regression line of the quarterly numbers of *seclusions* before 2006 differs significantly from that after 2006 (p < 0.001). The slopes of the regression line of the quarterly numbers of *involuntary medications* before and after 2006 do not differ significantly from one another.

The use of involuntary medication had increased by 8.5% annually from 1998 to 2005 (logit slope = 1,085). Between 2006 and 2009, this increase was 8.0% (logit slope = 1.080, difference −0,5%: z = −0.54, not significant) see Figure 1.

### Corrected numbers

A 3.3% annual decrease in the number of seclusions *per involuntary hospitalization* from 1998 to 2005 (logit slope = 0.967) was followed between 2006 and 2009 by a significantly greater annual decrease of 4.7% (logit slope = 0.953, difference −1,4%: z = −2.37, p = 0.018).

A 1.8% annual increase in the number of involuntary medications *per involuntary hospitalization* from 1998 to 2005 (logit slope = 1.018) became a significantly greater annual increase of 5.1% between 2006 and 2009 (logit slope = 1.051, difference 3,3%: z = 3.16, p = 0.002).

## Discussion

### Seclusions

After the Dutch governmental initiative in 2006, the earlier rise in numbers of seclusions was successfully changed into a decrease of 2% annually. A reduction occurred despite a rise in the number of involuntary hospitalizations between 1998 and 2009. However, this failed to meet the national goal of reducing the use of seclusion by 10% annually.

It may be that the decrease in seclusions could have been larger when the criteria for receiving the grant would have included specific guidelines. For example, despite the evidence on successful interventions for reducing the use of seclusion [6,7], there were no criteria on implementing evidence based- interventions which led to the use of many different interventions, mostly non evidence based. In addition not all hospitals and wards participated in the program.

### Involuntary medication

The faster increase in the use of involuntary medication after the government’s 2006 initiative was apparent only after correcting for the number of involuntary hospitalizations. Three possible explanations are 1) lower use of seclusion may have led to an increase in involuntary medication, 2) registration was better – which was a criterion for obtaining a seclusion-reduction grant or 3) the increase may have been an effect of a change in the Mental Health Act in 2008. Beforehand, involuntary treatment was only legally allowed to prevent ‘serious danger’ caused by psychiatric illness. In the new legal text the word ‘serious’ was deleted, which broadened the options for involuntary treatment.

### Other studies

Although the legal and regulatory context differs between countries, it is interesting to compare our results to other national studies about reducing seclusion. In the USA, for example, after state-wide policy changes, state leadership and other interventions, the Pennsylvania state hospital system successfully reduced the use of seclusion and restraint nearly to zero [8]. Similarly, after changes in the rules of the Health Care Financing Administration on seclusion and restraint, the use of these coercive measures also decreased in Rochester, New York (adults and children) [9], and Middletown, Connecticut (children and adolescents)[10]. In Finland, however, even after several legislative changes, the risk of seclusion and mechanical restraint did not decrease over a 15-year period [11]. But not only did these legislative changes not explicitly restrict the use of seclusion and restraint, they were implemented without any national educational program or practical guidelines. As in the present study, it may be that these legislative changes would have yielded better results when there would have been national guidelines as the authors indicate.

### Limitations

Due to the observational design of this study, we cannot say whether the changes observed in use of the coercive measures were caused by the governmental initiative.

The use of seclusion and involuntary medication may have been underreported to the DHCI [2], especially before 2006. Better registration after 2006, however, would mean that the results of the nationwide program have been underestimated. Moreover, we only had access to national aggregated numbers on coercive measures, and therefore we could not compare the effect of different strategies for reducing seclusion between hospitals or interventions.

Since no data were available on the duration of seclusion, we could not assess the effects of the nationwide program on the length of seclusion.

The definition of involuntary medication leaves some room for interpretation, since sometimes the border between persuasion and coercion may be floating. This may have caused underreporting of the use of involuntary medication.

Finally, only four years of follow-up were available. A longer follow-up period may be needed to detect longer-term changes in the use of coercive measures.

## Conclusions

After the start of the nationwide program the numbers of seclusion reduced by 2% annually, a modest reduction that did not meet the goal of a 10% annual decrease. The number of involuntary medications did not change; instead, after correction for the number of involuntary hospitalizations, it increased. These results may be underlain by the absence of national guidelines for reducing coercive interventions.

Fur future nationwide programs we recommend use of more extensive national guidelines, especially in implementing and monitoring evidence based programs for reducing the use of seclusion and other coercive measures, and involvement of all relevant wards nationwide.

## Abbreviation

DHCI: Dutch Health Care Inspectorate.

## Competing interests

The authors declare that they have no competing interests.

## Authors’ contributions

The conception, design, and interpretation later on, was performed by CM, HN, EN and FV. CM contacted the Dutch Health Care Inspectorate for providing the data. EN provided information on the nationwide program, as he is involved as head of research of this program. FV and EN restructured the data and performed the analyses together with the statistician, DU. DU advised closely on the appropriate method. FV wrote the first and following drafts, while CM, HN and EN revised the drafts critically for important intellectual content. CM primarily re-edited most of the drafts. All authors approved the final version.

## Pre-publication history

The pre-publication history for this paper can be accessed here:

http://www.biomedcentral.com/1471-244X/12/231/prepub

## References

[B1] Raboch J Kalisova L Nawka A Kitzlerova E Onchev G Karastergiou A Magliano L Dembinskas A Kiejna A Torres-Gonzales F Use of coercive measures during involuntary hospitalization: findings from ten European countries Psychiatr Serv 2010 61 10 1012 1017 10.1176/appi.ps.61.10.1012 20889640

[B2] Janssen WA Noorthoorn EO de Vries WJ Hutschemeakers GJ Lendemeijer HH Widdershoven GA The use of seclusion in the Netherlands compared to countries in and outside Europe Int J Law Psychiatry 2008 31 6 463 470 10.1016/j.ijlp.2008.09.002 18954906

[B3] Steinert T Lepping P Bernhardsgrutter R Conca A Hatling T Janssen W Keski-Valkama A Mayoral F Whittington R Incidence of seclusion and restraint in psychiatric hospitals: a literature review and survey of international trends Soc Psychiatry Psychiatr Epidemiol 2010 45 9 889 897 10.1007/s00127-009-0132-3 19727530

[B4] College Tarieven gezondheidszorg zorgautoriteit Policy rule coercion and compulsion [Beleidsregel dwang en drang]. In., vol. CA-65 2005

[B5] Mulder CL Uitenbroek D Broer J Lendemeijer B van Veldhuizen JR van Tilburg W Lelliott P Wierdsma AI Changing patterns in emergency involuntary admissions in the Netherlands in the period 2000–2004 Int J Law Psychiatry 2008 31 4 331 336 10.1016/j.ijlp.2008.06.007 18667238

[B6] Scanlan JN Interventions to reduce the use of seclusion and restraint in inpatient psychiatric settings: what we know so far a review of the literature Int J Soc Psychiatry 2010 56 4 412 423 10.1177/0020764009106630 19617275

[B7] Gaskin CJ Elsom SJ Happell B Interventions for reducing the use of seclusion in psychiatric facilities: review of the literature Br J Psychiatry 2007 191 298 303 10.1192/bjp.bp.106.034538 17906239

[B8] Smith GM Davis RH Bixler EO Lin HM Altenor A Altenor RJ Hardentstine BD Kopchick GA Pennsylvania State Hospital system’s seclusion and restraint reduction program Psychiatr Serv 2005 56 9 1115 1122 10.1176/appi.ps.56.9.1115 16148327

[B9] Currier GW Farley-Toombs C Datapoints: use of restraint before and after implementation of the new HCFA rules Psychiatr Serv 2002 53 2 138 10.1176/appi.ps.53.2.138 11821540

[B10] Donovan A Plant R Peller A Siegel L Martin A Two-year trends in the use of seclusion and restraint among psychiatrically hospitalized youths Psychiatr Serv 2003 54 7 987 993 10.1176/appi.ps.54.7.987 12851435

[B11] Keski-Valkama A Sailas E Eronen M Koivisto AM Lonnqvist J Kaltiala-Heino R A 15-year national follow-up: legislation is not enough to reduce the use of seclusion and restraint Soc Psychiatry Psychiatr Epidemiol 2007 42 9 747 752 10.1007/s00127-007-0219-7 17598058

